# Characterizing Twitter Content About HIV Pre-exposure Prophylaxis (PrEP) for Women: Qualitative Content Analysis

**DOI:** 10.2196/43596

**Published:** 2023-05-11

**Authors:** Shimrit Keddem, Aneeza Agha, Sabrina Morawej, Amy Buck, Peter Cronholm, Sarita Sonalkar, Matthew Kearney

**Affiliations:** 1 Department of Family Medicine & Community Health University of Pennsylvania Philadelphia, PA United States; 2 Center for Health Equity, Research & Promotion Corporal Michael J Crescenz VA Medical Center US Department of Veterans Affairs Philadelphia, PA United States; 3 Center for Public Health Perelman School of Medicine University of Pennsylvania Philadelphia, PA United States; 4 Leonard Davis Institute for Health Economics University of Pennsylvania Philadelphia, PA United States; 5 Division of Family Planning, Department of Obstetrics and Gynecology Perelman School of Medicine University of Pennsylvania Philadelphia, PA United States

**Keywords:** HIV pre-exposure prophylaxis, women, Twitter, social media, health communication, communication, HIV, barrier, awareness, tweets, application, prevention

## Abstract

**Background:**

HIV remains a persistent health problem in the United States, especially among women. Approved in 2012, HIV pre-exposure prophylaxis (PrEP) is a daily pill or bimonthly injection that can be taken by individuals at increased risk of contracting HIV to reduce their risk of new infection. Women who are at risk of HIV face numerous barriers to HIV services and information, underscoring the critical need for strategies to increase awareness of evidence-based HIV prevention methods, such as HIV PrEP, among women.

**Objective:**

We aimed to identify historical trends in the use of Twitter hashtags specific to women and HIV PrEP and explore content about women and PrEP shared through Twitter.

**Methods:**

This was a qualitative descriptive study using a purposive sample of tweets containing hashtags related to women and HIV PrEP from 2009 to 2022. Tweets were collected via Twitter’s API. Each Twitter user profile, tweet, and related links were coded using content analysis, guided by the framework of the Health Belief Model (HBM) to generate results. We used a factor analysis to identify salient clusters of tweets.

**Results:**

A total of 1256 tweets from 396 unique users were relevant to our study focus of content about PrEP specifically for women (1256/2908, 43.2% of eligible tweets). We found that this sample of tweets was posted mostly by organizations. The 2 largest groups of individual users were activists and advocates (61/396, 15.4%) and personal users (54/396, 13.6%). Among individual users, most were female (100/166, 60%) and American (256/396, 64.6%). The earliest relevant tweet in our sample was posted in mid-2014 and the number of tweets significantly decreased after 2018. We found that 61% (496/820) of relevant tweets contained links to informational websites intended to provide guidance and resources or promote access to PrEP. Most tweets specifically targeted people of color, including through the use of imagery and symbolism. In addition to inclusive imagery, our factor analysis indicated that more than a third of tweets were intended to share information and promote PrEP to people of color. Less than half of tweets contained any HBM concepts, and only a few contained cues to action. Lastly, while our sample included only tweets relevant to women, we found that the tweets directed to lesbian, gay, bisexual, transgender, queer (LGBTQ) audiences received the highest levels of audience engagement.

**Conclusions:**

These findings point to several areas for improvement in future social media campaigns directed at women about PrEP. First, future posts would benefit from including more theoretical constructs, such as self-efficacy and cues to action. Second, organizations posting on Twitter should continue to broaden their audience and followers to reach more people. Lastly, tweets should leverage the momentum and strategies used by the LGBTQ community to reach broader audiences and destigmatize PrEP use across all communities.

## Introduction

HIV remains a persistent health problem in the United States, with almost 37,000 new diagnoses in 2019 [1]. Women are more vulnerable to HIV infection than heterosexual men and make up 19% of new infections [2]. Women who are at risk of HIV face numerous barriers to HIV services and information, underscoring the critical need for strategies to increase awareness of evidence-based HIV prevention methods among women, with particular attention to at-risk women.

HIV pre-exposure prophylaxis (PrEP) is a daily pill or bimonthly injection that can be taken by individuals at increased risk of contracting HIV to reduce their risk of new infection. Approved by the US Food and Drug Administration in 2012, PrEP is an effective and integral tool in ending the HIV epidemic. Despite a decade having passed since its approval, serious disparities still exist in PrEP uptake, especially among women. While women make up a significant portion of newly infected people, only 10% of eligible women are prescribed PrEP [3].

Increasing awareness of HIV PrEP is a vital first step to bolstering uptake. However, awareness of HIV PrEP among US women is low [4] despite high levels of interest [5,6]. In a nationally representative sample of US men and women, only 22% of PrEP-eligible women in the United States were aware of PrEP as an HIV prevention option [4]. Women’s low level of awareness of HIV PrEP has been linked to the lack of PrEP advertising in the places where women seek health care [7]. When presented with the option of PrEP for HIV prevention, a majority of eligible women indicated being likely to use it [6]. Because women’s interest in PrEP may be complicated by sociocultural factors, such as stigma and medical distrust [8], mass media can be a powerful and effective tool for delivering information about PrEP [9,10].

Social media has emerged as an important source of information about PrEP and a space where people share health information and advice. Twitter is a platform containing a rich source of information, where researchers can monitor public perceptions and opinions about health services, medications, and treatments [11]. About 1 in 5 US adults use Twitter [12]. Because Twitter can be anonymous and far reaching, it provides the researcher with an important perspective on health communication to understand what is available and being circulated to the public about PrEP and what is being targeted directly at women.

Health communication studies about PrEP content directly targeted at women on social media have been limited [13]. A few studies have examined PrEP-related perceptions, trends, and social representations on Twitter in general, indicating an overall positive sentiment about PrEP [14] and a potential to counter stigmatizing narratives about PrEP [15]. To our knowledge, no studies have looked specifically at content that is directly related to PrEP targeted specifically at women on Twitter.

This study aimed to identify historical trends in the use of Twitter hashtags specific to women and HIV PrEP and explore content about women and PrEP shared through Twitter. In addition, we examined characteristics of linked content, user engagement, and theoretical constructs from the Health Belief Model (HBM) present in identified tweets.

## Methods

### Ethical Considerations

As this work involved publicly available Twitter data and did not include human-subjects research, it was exempt from ethical board review.

### Data Collection and Sampling

**Figure 1:**
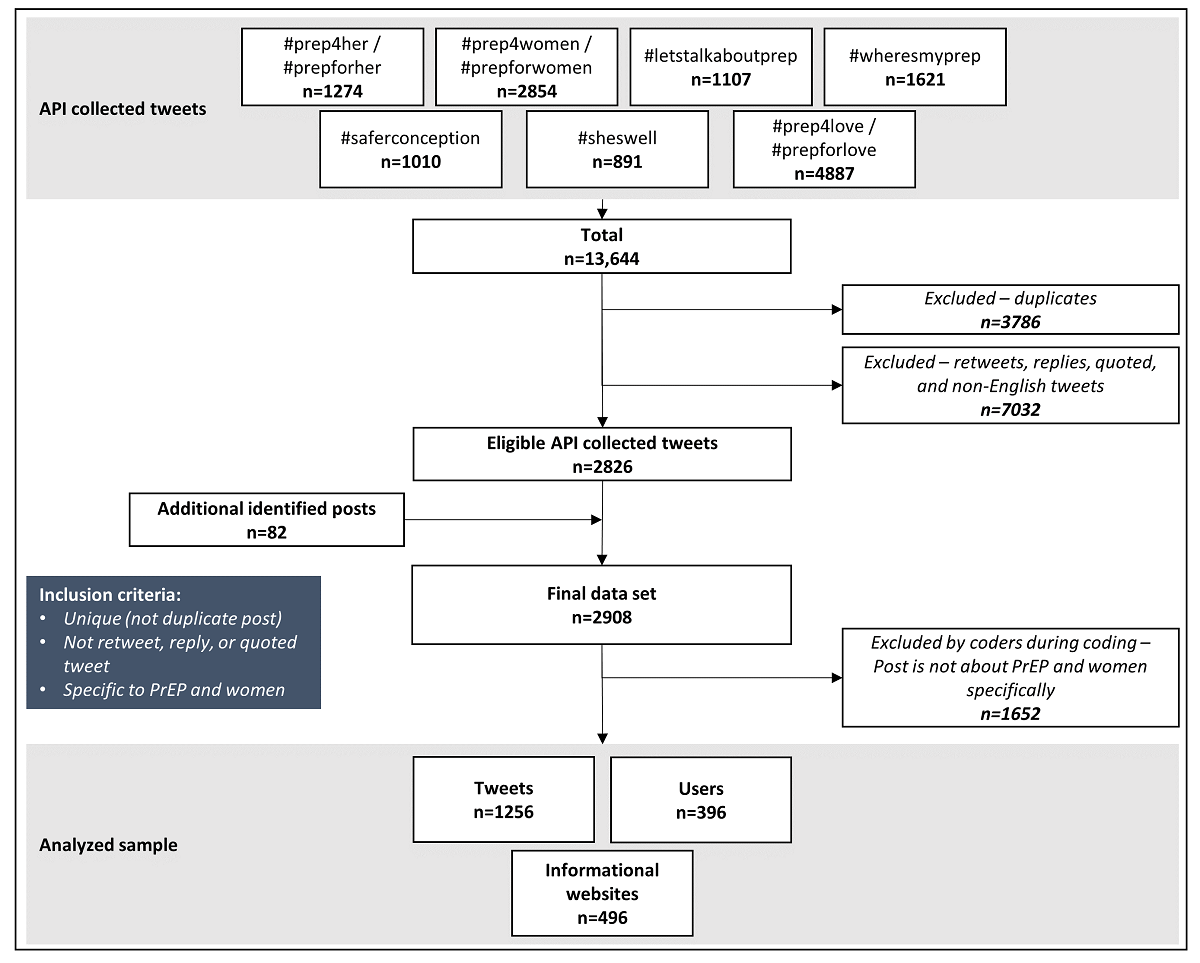
Data collection and sampling. All tweets collected were created between June 3, 2009, and April 14, 2022. API: application programming interface; PrEP: pre-exposure prophylaxis.

In this descriptive study, we explored Twitter posts about HIV PrEP related to and directly targeting women. All available tweets were collected via Twitter’s application programing interface (API) using the *academictwitteR* package in R Studio (R Foundation for Statistical Computing) [16]. The Twitter API allows access to public tweets from July 15, 2006—Twitter’s launch date. Although we collected data from Twitter’s launch through April 14, 2022, the earliest relevant tweet was not created until May 7, 2014. We used an iterative approach to identify the most appropriate hashtags for purposive sampling. We first reviewed a sample of 100 tweets using keywords including “HIV,” “women,” “woman,” “girl,” “girls,” “PrEP,” “female,” and “females” to find the most relevant hashtags used in content that was specifically related to women or social media campaigns directed at women. Once these were identified, we used these hashtags to query the Twitter API and create an initial subsample of 175 tweets used for codebook development (see below). In the process of codebook development and throughout early analysis, as new hashtags were identified, they were added to the sample. Through this approach, we collected all public tweets with the following hashtags specific to women and PrEP: #prep4her, #prepforher, #prep4women, #prepforwomen, #sheswell, #prep4love, #prepforlove, #letstalkaboutprep, #wheresmyprep, and #saferconception (n=13,644). We excluded duplicates (n=3786), retweets, replies, quoted tweets, and non-English tweets (n=7032), for a final sample of 2826 eligible tweets. When coding began, tweets identified through our coding that did not mention any of the hashtags but were noted to be content relevant (n=82) were added. For example, some tweets in our original data set using the above hashtags were linked to new tweets about women and PrEP that did not contain the above hashtags. Through this approach, we created a final sample of 2908 eligible tweets from 808 eligible users. Each tweet was then reviewed during coding to determine if it was relevant or not (see the Tweet Coding section below). Figure 1 summarizes our data collection and sampling.

### Qualitative Analysis

#### Codebook Development and Theoretical Framework

The codebook (Multimedia Appendix 1) was developed by a team of 3 researchers (SK, AA, and MK) with expertise in qualitative methods, social media, and public health; it included the concepts of the HBM [17]. The HBM asserts that health messaging will be successful if it appropriately targets perceived barriers, benefits, self-efficacy, and threat [18]. Because the HBM can be used to guide health promotion and disease prevention efforts, we used the constructs of the model in our coding and analysis to assess the quality of the information being disseminated about PrEP to women on Twitter. The codebook contained 5 theoretical constructs, including risk of HIV/AIDS, benefits of PrEP, self-efficacy and empowerment, cues to action, and barriers to PrEP use. Using thematic saturation as a guidepost to completion of codebook development [19], we reviewed data until no new concepts emerged. Throughout codebook development, a total of 175 tweets (6% of the sample of 2908) were reviewed by the team and coded to test the codebook’s functionality. Discrepancies in coding during codebook development were resolved with team discussions and revisions of the codebook before proceeding with coding of the rest of the sample.

#### Tweet Coding

**Figure 2:**
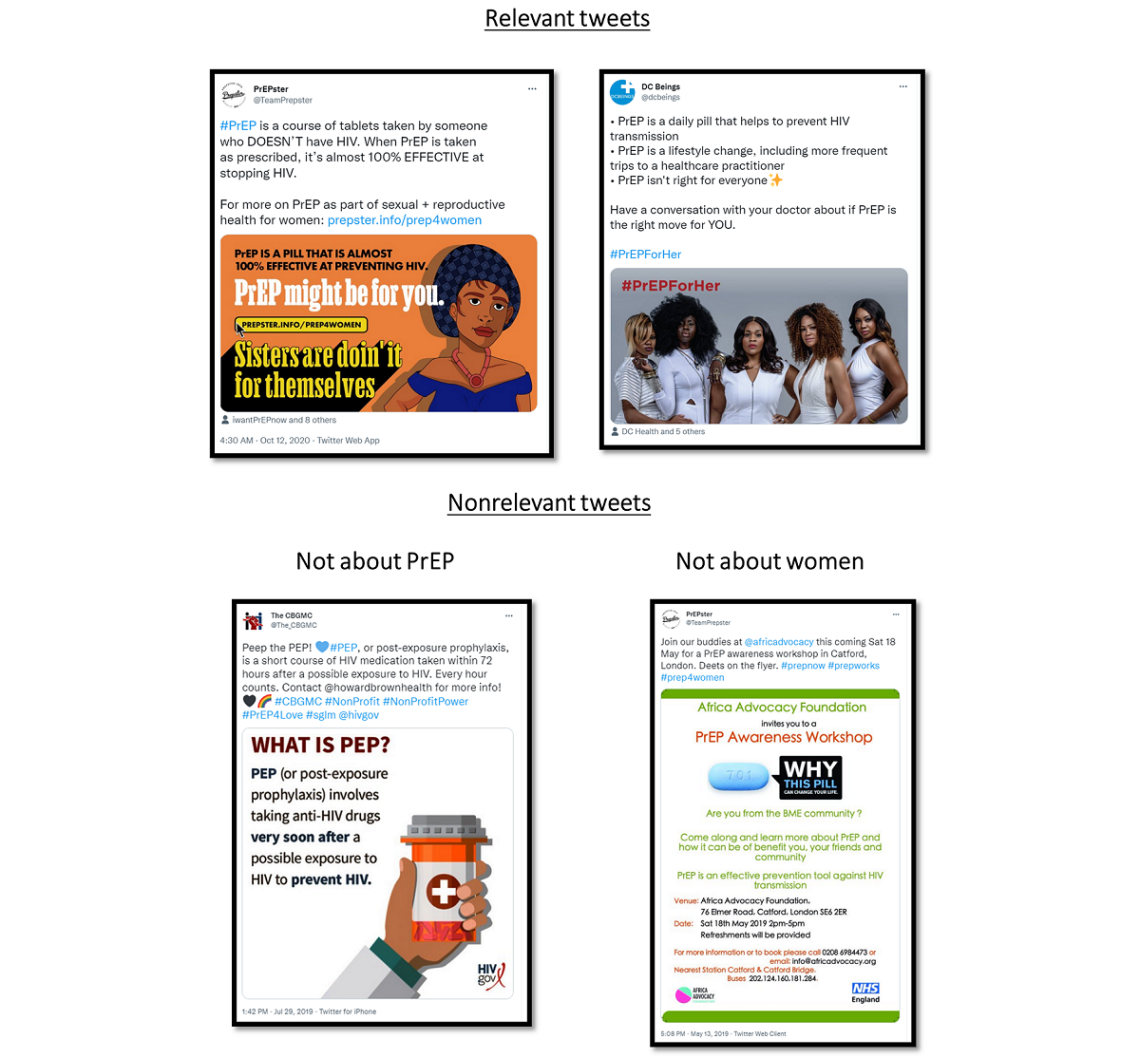
Relevant versus nonrelevant tweets. PrEP: pre-exposure prophylaxis.

As the first step in the coding process, all coders reviewed each tweet for its relevance to women and to PrEP. Tweets that were not relevant to women or PrEP or that did not include enough information were excluded from the sample. Figure 2 shows examples of relevant versus nonrelevant tweets. Of the sample of 2908 eligible tweets, a total of 1256 (43.2%) from 396 unique users were coded as relevant to our study focus of content about PrEP specifically for women. For each tweet, coders identified the presence of videos, imagery, links to external websites, and links to other tweets (included or not included in the sample). Images and videos were coded for the number and type of people present, the presence of symbolism or iconography, a logo or brand, marketing or promotional materials, a web address, contact information, a health care setting, local scenery, or a campaign slogan. Each tweet was coded for constructs of the HBM, including risk of HIV, benefits of PrEP, self-efficacy or empowerment, cues to action, and barriers to PrEP. In addition, each tweet was coded for its purpose and the perceived target audience. The target audience or audiences were identified based on any specific population mentioned in the tweet or images included in the tweet. Tweets that targeted multiple audiences were coded to as many audiences as were applicable. Transgender audiences were coded only if explicitly noted; otherwise, all content pertaining to women was coded as cisgender. Links within tweets that were categorized as informational websites were followed and fully coded using the same tweet codebook.

#### User Coding

A separate codebook was developed for user information. User type was stratified into 2 broad groups with subcategories: individual (ie, self-identified activists, health professionals, and researchers) or organizational (ie, nonprofit or health care organizations). When provided, we also coded for user location at the country level. After determining that most users originated in the United States, we also documented specific localities or states, if that information was provided. For individual Twitter users, we coded perceived gender, sexual orientation, and race based on self-reported text descriptions and user profile images. “Black” or “Hispanic” were selected only if users explicitly self-identified with these terms; otherwise, “person of color” was selected based on profile pictures. If the user did not self-identify, coders documented their perceptions of user characteristics.

#### Coding Process

Following codebook development, the full sample of tweets was coded by a group of 5 coders (SK, AA, SM, AB, and MK) using Qualtrics (Qualtrics International Inc). A total of 20% of the sample (n=578) was coded by at least 2 team members to ensure interrater reliability as measured based on percentage agreement [20]. Discrepancies were resolved by group consensus; final agreement scores in each domain were as follows: video content (mean 83%, SD 0.135%), link content (mean 97%, SD 0.032%), image attributes (mean 91%, SD 0.076%), HBM constructs (mean 82%, SD 0.110%), tweet purpose (mean 88%, SD 0.104%), target audience (mean 88%, SD 0.089%), and people shown (mean 84%, SD 0.073%). Following a review of discrepancies, 2 team members (AA and SK) completed coding of users who created relevant tweets and 2 team members (AA and SM) coded informational website characteristics.

### Statistical Analysis

Descriptive statistics were generated for the coded characteristics of relevant tweets, informational websites, and users, as well as for associated metadata (eg, followers and retweets). All characteristics were coded as binary or categorical variables. We also created an aggregate variable of audience engagement comprising total retweets, likes, and quotes. For tweets, we calculated mean audience engagement values for selected tweet content codes. We then used exploratory factor analysis of coded tweet text and image characteristics to identify latent thematic dimensions or factors across the different characteristics of tweets. Factors with eigenvalues (λ) greater than or equal to 1.0 were retained. We determined characteristics with factor loadings greater than or equal to 0.25 to be salient. All statistical analyses were conducted in Stata/SE (version 15.1; StataCorp LLC).

## Results

### Sample Characteristics

Table 1 presents descriptive statistics for all relevant tweets (ie, audience, HBM construct or constructs, link contents, and purpose). The tweets received 3.5 (SD 12.5, median 1, max 277) retweets and 7.3 (SD 39.5, median 2, max 1112) likes. The total average engagement rate (retweets plus quotes plus likes) was 16.1 (SD 59.0, median 5, max 1360) engagements per tweet. Users averaged 9841.2 (SD 49,796.6, median 1336.5) followers and followed 3080.7 (SD 29,551, median 898.5) other accounts. The earliest relevant tweet in our sample was posted on May 7, 2014; more than 60% of Tweets were posted in 2016, 2017, and 2018 (25.2%, 16.9%, and 22.3%, respectively). Table 2 presents a timeline of tweets from 2014 to 2022.

**Table 1:** Tweet content characteristics shown as univariate and bivariate descriptive statistics for relevant tweets (n=1256). Engagement is an aggregate measure that includes total quotes, retweets, and likes.

Category/characteristics		Tweets, n (%)	Tweet engagement	
			Median	Mean (SD)
**Audience**				
	Cisgender women	1023 (81.4)	4	13.4 (38.0)
	Black people	304 (24.2)	4	13.1 (29.0)
	People of color	276 (22)	5	16.0 (35.0)
	None/undetermined	102 (8.1)	5	14.0 (21.8)
	Transgender women	108 (8.6)	6	29.6 (94.5)
	Health professionals	103 (8.2)	5	14.4 (31.8)
	Heterosexual couples	94 (7.5)	4	10.6 (21.6)
	Lesbian, gay, bisexual, transgender, queer	68 (5.4)	7	37.5 (116.3)
	Serodiscordant couples	64 (5.1)	3	4.8 (7.1)
**Health Belief Model**				
	Benefits	395 (31.4)	4	14.8 (52.3)
	Self-efficacy	281 (22.4)	4	12.5 (25.8)
	Risk of HIV/AIDS	176 (14)	4	9.6 (20.2)
	Barriers to PrEP^a^	82 (6.5)	4	15.2 (26.6)
	Cues to action	63 (5)	5	11.8 (18.6)
**Purpose**				
	Promoting PrEP use and access	760 (60.5)	5	14.6 (42.6)
	Sharing information/resources	700 (55.7)	4	12.5 (25.7)
	Providing event details	205 (16.3)	5	10.8 (15.6)
	Describing personal narrative/experience	79 (6.3)	3	8.9 (16.0)
	Raising PrEP awareness	78 (6.2)	4	14.5 (34.0)
**Image type**				
	Image plus text	606 (48.2)	6	18.7 (47.6)
	Tweet only	548 (43.6)	6	7.3 (4.2)
	Video	99 (7.9)	4	14.8 (27.4)
	Image only	3 (0.2)	3	7.2 (13.8)
**Imagery**				
	Person present	657 (92.8)	5	17.7 (46.2)
	Photograph	535 (75.6)	6	16.2 (29.2)
	None	333 (47)	3	7.6 (15.0)
	Campaign slogan	145 (20.5)	6	18.0 (33.5)
	PrEP pill/medication	126 (17.8)	4	17.3 (83.1)
	Marketing/promotional materials	106 (15)	15.5	27.4 (33.8)
	Other	103 (14.5)	4.5	11.9 (20.4)
	Conference presentations and materials	84 (11.9)	6	12.4 (15.6)
	Web address URL	80 (11.3)	5.5	19.5 (35.1)
**Person/people present**				
	Female	639 (97.3)	5	17.5 (46.5)
	Person of color	464 (70.6)	5	18.6 (52.5)
	Male	290 (44.1)	6	17.2 (30.2)
	Black	287 (43.7)	5	15.0 (28.6)
	White	264 (40.2)	6	20.4 (33.5)
	Male plus female	244 (37.1)	6	17.0 (31.2)
	Other gender	70 (10.7)	5	32.4 (114.2)
	Hispanic	62 (9.4)	4.5	19.1 (41.0)
**Other person characteristics**				
	Cartoon character/mascot	133 (20.2)	4.5	17.2 (28.3)
	Health care worker	123 (18.7)	5	15.9 (28.2)
	Lesbian, gay, bisexual, transgender, queer	114 (17.4)	6	31.1 (92.5)
	Other	82 (12.5)	5	14.5 (18.1)
	Journalist	46 (7)	5.5	16.2 (22.4)

^a^PrEP: pre-exposure prophylaxis.

**Table 2:** Timeline of relevant tweets from 2014 to 2022 (n=1256).

Year	Tweets, n
2014	3
2015	305
2016	204
2017	269
2018	113
2019	61
2020	106
2021	99

### Tweet Contents and Engagement

Most relevant tweets were directed toward a target audience of cisgender women (1023/1256, 81.4%). One-third were directed toward people of color (429/1256, 34.2%), including almost a quarter specifically featuring people who are Black (304/1256, 24.2%). Nearly half of tweets (618/1256, 49.2%) contained HBM constructs, such as promoting the benefits of PrEP (395/1256, 31.4%) or self-efficacy to use PrEP (281/1256, 22.4%). Only 5% (63/1256) of tweets contained cues to action. The main purpose of most tweets was to promote PrEP use and access (760/1256, 60.5%) or sharing of information and resources (700/1256, 55.7%). We also observed that tweets had among the highest levels of engagement from audiences when they were directed toward transgender women (29.6 engagements/tweet versus 16.1 average) and lesbian, gay, bisexual, transgender or queer (LGBTQ) individuals (37.5 engagements/tweet). Engagement with tweets was stronger when tweets contained imagery or videos, had a person present in the image, and contained a campaign slogan.

Table 1 presents descriptive statistics for imagery shown in relevant tweets. Nearly half of tweets featured both image and text content (606/1256, 48.2%), whereas there were fewer tweets containing just text (548/1256, 43.6%). Videos were also shown in some tweets (99/1256, 7.9%). Of the 708 tweets featuring any form of visualization—image with text, image only, or video—nearly all showed people (657/708, 92.8%), including cartoons (133/657, 20.2%), and many showed health care workers (123/657, 18.7%). When people were shown, nearly all were women (639/657, 97.3%) and people of color (464/657, 70.6%). Other aspects of visualizations included campaign slogans (145/708, 20.5%), PrEP medication (126/708, 17.8%), campaign marketing materials (eg, T-shirts; 106/708, 15%), and conference presentations or materials (84/708, 11.9%).

### User Attributes

Of the 396 unique users who created relevant tweets in our sample, most were organizations rather than individuals (230/396, 58.1% and 166/3964, 1.9%, respectively; Table 3). Advocacy groups and nonprofit organizations comprised the largest user group (120/396, 30.3%), followed by health care organizations (59/396, 14.9%). The 2 largest groups of individual users were activists and advocates (61/396, 15.4%) and personal users (54/396, 13.6%). Among individual users, most were female (100/164, 61%). Users’ race and ethnicity were relatively evenly split between White people (63/164, 39.9%) and people of color (61/164, 38.6%), and a further 6.3% (10/164) identified as Black. Most tweets originated from the United States (256/396, 64.6%), followed by the United Kingdom (88/396, 22.2%). The largest groups of US-based tweets originated from 3 major cities: Washington, DC (44/256, 17.2%); New York (29/256, 11.3%); and Chicago (25/256, 9.8%). Most tweets came from a relatively small number of geographic locations and were associated with specific local PrEP campaigns (eg, PrEP4Love).

**Table 3:** Coded attributes for users of relevant tweets (n=396 users).

Category/characteristics				Users, n (%)
**User type**				
	**Individual**		166 (41.9)	
		Activist/advocate	61 (15.4)	
		Personal/other	54 (13.6)	
		Health professional	30 (7.6)	
		Researcher/academic	21 (5.3)	
	**Organization**		230 (58.1)	
		Advocacy/nonprofit organization	120 (30.3)	
		Health care	59 (14.9)	
		General/other	51 (12.9)	
**Gender (n=164)**				
		Female	100 (61)	
		Male	45 (27.4)	
		Other	16 (9.8)	
**Other characteristics (n=164)**				
		Personal account	142 (86.6)	
		HIV/AIDS work	49 (29.9)	
		Professional account	41 (25)	
		Lesbian, gay, bisexual, transgender, and queer or questioning	38 (23.2)	
**Race/ethnicity (n=164)**				
		White	63 (38.4)	
		Person of color	61 (37.1)	
		Other/undetermined	22 (13.4)	
		Black	10 (6.1)	
**Location**				
	**United States**		256 (64.6)	
		Washington, DC (eg, PrEP4Her campaign)	44 (17.2)	
		New York	29 (11.3)	
		Chicago	25 (9.8)	
		United States in general (eg, Centers for Disease Control and Prevention or National Institutes of Health)	16 (6.3)	
		San Francisco	11 (4.3)	
	United Kingdom		88 (22.2)	
	Other		34 (8.6)	
	None provided/undeterminable		18 (4.5)	

### Website Link Contents

Of the 820 tweets that contained website links, most were for informational websites (496/820, 60.5%; Table 4). We observed similar characteristics in linked websites as in tweets themselves. The websites featured HBM elements, including PrEP’s benefits (75/281, 26.7%) and self-efficacy (35/281, 12.5%), as well as cues to action for obtaining PrEP (43/281, 15.3%). One-third of websites had the main purpose of either sharing resources and information (94/281, 33.5%) or promoting PrEP use and access (85/281, 30.2%), while nearly 1 in 5 featured PrEP users’ personal narratives or experiences (47/281, 16.7%). We observed a more diverse range of intended user audiences for information websites. The most common audience was cisgender women (82/281, 29.2%). However, many tweets also targeted any or all the following: people of color (35/281, 12.5%), heterosexual couples (35/281, 12.5%), serodiscordant couples (31/281, 11%), and pregnant women (26/281, 9.3%).

**Table 4:** Link characteristics and contents. A total of 820 of the 1256 (65.3%) relevant tweets contained links. Additional characteristics are presented for linked informational websites.

Category/characteristics					Values, n (%)
**Link contents**					
	Informational website			496 (60.5)	
	Tweets from another user (shared—not retweets)			140 (17.1)	
	News article			96 (11.7)	
	Research article			68 (8.3)	
	Broken link			69 (8.4)	
	Instagram/Facebook post			42 (5.1)	
**Linked informational websites** **(n=281)**					
	**Health Belief Model**				
		Benefits of PrEP^a^	75 (26.7)		
		Cues to action	43 (15.3)		
		Self-efficacy/empowerment	35 (12.5)		
		Risk of HIV/AIDS (ie, susceptibility, severity)	31 (11)		
	**Purpose**				
		Sharing information/resources	94 (33.5)		
		Promoting PrEP use and access	85 (30.2)		
		Describing personal narrative/experience	47 (16.7)		
		Raising awareness of PrEP	28 (10)		
		Soliciting audience engagement	24 (8.5)		
	**Audience**				
		Women (cisgender)	82 (29.2)		
		Other	37 (13.2)		
		People of color	35 (12.5)		
		Heterosexual couples	35 (12.5)		
		Serodiscordant couples	31 (11)		
		Pregnant women	26 (9.3)		
		Transgender women	21 (7.5)		
		Health professionals	17 (6)		

^a^PrEP: pre-exposure prophylaxis.

### Factor Analysis

Table 5 presents a factor analysis for select characteristics of relevant tweets. We retained 3 factors accounting for 70.1% of variance, loaded with 15 coded characteristics for link contents, HBM constructs, purpose, and audience. The first factor accounted for 37.8% of variance (λ=2.88) and included tweets sharing resources and information and promoting PrEP’s benefits, use, and access; they were targeted toward cisgender women, people who are Black, and other people of color. The second factor accounted for 18.3% of variance (λ=1.4) and included tweets appealing to a diverse range of audiences, including heterosexual and serodiscordant couples, transgender women, and LGBTQ people. The third factor accounted for 14% of variance (λ=1.07) and included tweets highlighting the risk of HIV/AIDS but without a clear intended audience.

**Table 5:** Factor analysis items and factor loadings. All factors retained had eigenvalues (λ) greater than or equal to 1.0. All items retained had factor loadings greater than or equal to 0.25.

Items			Component factor loadings				
			1		2		3
**Link contents**							
	Informational website	0.53		—^a^		—	
**HBM^b^**							
	Benefits	0.53		—		—	
	Self-efficacy	0.41		—		—	
	Cues to action	0.32		—		—	
**Purpose**							
	Promoting PrEP^c^ use and access	0.49		—		—	
	Sharing information/resources	0.62		—		—	
**Audience**							
	Women (cisgender)	0.40		—		—	
	People of color	0.35		—		—	
	Black people	0.36		—		—	
	Heterosexual couples	—		0.38		—	
	Lesbian, gay, bisexual, transgender, queer	—		0.31		—	
	Serodiscordant couples	—		0.33		—	
	Transgender women	—		0.29		—	
	None/undetermined	—		—		0.45	
**HBM**							
	Risk of HIV/AIDS	—		—		0.25	

^a^Not applicable.

^b^HBM: Health Belief Model.

^c^PrEP: pre-exposure prophylaxis.

## Discussion

### Principal Findings

This study describes tweets about HIV PrEP directed at trans- and cisgender women in a global sample over a nearly 10-year period. We found that this sample of tweets was posted by a relatively small group of users (mostly organizations) and significantly decreased after 2018. Most tweets specifically targeted people of color, including imagery and links to informational websites. Less than half of tweets contained any HBM concepts, and only a few contained cues to action. Lastly, while our sample included only tweets relevant to women, we found that the tweets directed to LGBTQ audiences received the highest levels of audience engagement.

Our finding that few tweets contained theoretical constructs is consistent with evidence that theory remains underused in social marketing campaigns [21,22]. Health behavior frameworks are foundational to developing health promotion campaigns [23,24]. Use of theory can help in understanding the target audience and specific segments of the population and in message framing that encourages action and engagement. It is critical for future social media messaging about PrEP directed at women to incorporate theoretical frameworks in a thoughtful and systematic way [22].

The extensive use of tweets using inclusive imagery targeted at women of color is an appropriate approach for more effective engagement. In addition to inclusive imagery, our factor analysis indicated that more than a third of tweets were intended to share information and promote PrEP to people of color. Use of inclusive imagery is a guiding principle of health communication, because representation affects how people construct identity and develop normative behaviors [25,26]. One barrier driving Black women’s experiences in accessing and receiving sexual health care is rooted in stigma-based fear of judgment linked to racial stereotypes about sex, sexuality, and at-risk behaviors [27]. Inclusive and positive imagery of women of color is central to creating a more holistic representation of sexual health that normalizes and destigmatizes prevention methods such as HIV PrEP.

We found that a relatively small sample of Twitter users, mostly organizations, generated content over a decade-long period. This is consistent with findings that health organizations tend to follow each other in relatively small networks on social media and tend to circulate the same information to the same followers [28]. In addition, a significant portion of tweets in our sample originated from a few localized PrEP campaigns. These included a few robust campaigns, such as the PrEP4Love campaign [10] in Chicago and Prepster [29] in the United Kingdom. Content from these campaigns was inclusive of many racial and ethnic groups and gender identities and included powerful imagery, comprehensive informational resources, and memorable slogans. However, given the small number of users engaged, this rich content did not spread beyond this isolated network. This highlights an opportunity for organizations to continue to pursue avenues to reach a wider and more diverse audience.

Audience engagement with tweets that were directed to LGBTQ individuals highlights both the disparity in access to PrEP for women and an opportunity for improvement. In the community of men who have sex with men (MSM), PrEP use has proliferated since its approval in 2012. In that time, HIV PrEP among MSM has been significantly destigmatized, with PrEP use among the MSM community being considered a social norm. For example, in one study of a group of MSM, participants described the gay community coalescing around the use of PrEP as individuals taking responsibility to protect themselves and others against HIV [30]. Organizations and individuals wishing to eliminate the disparity in PrEP access for women may want to leverage the momentum created in MSM communities and study messaging approaches to create and disseminate social media content.

The drop in the number of tweets about PrEP directed at women since 2018 is a concerning trend. PrEP received national attention in the United States with the release of new Centers for Disease Control and Prevention guidelines for high-risk individuals in 2014, which were subsequently updated in 2017. Guidelines were not updated again until 2021 with recommendations for greater inclusivity in offering PrEP. In the United Kingdom, PrEP received major publicity when it was announced in 2016 that PrEP would be made freely available to those at risk for HIV. However, attention for PrEP in the media declined after 2017, as is reflected in the patterns seen from the tweets in this sample. Moreover, the COVID-19 pandemic was a distraction from persistently high rates of HIV infection, which stayed constant throughout the pandemic, especially among young women [31]. Lastly, women continue to be excluded from HIV PrEP research, especially in the context of Descovy (tenofovir, alafenamide, and emtricitabine), a newer HIV-prevention drug that has proven safer for kidney and bone health [32] but has not been tested for vaginal or frontal exposure [33].

### Limitations

Several limitations should be considered in the interpretation of these results. Our sample was gathered based on Twitter hashtags, and it is possible that some hashtags related to women and PrEP were missed. However, as new hashtags were identified, they were added to the sample. In addition, our sample was limited to the Twitter platform, but there are several other social media platforms that include content about women and PrEP (eg, Instagram and YouTube). Our analyses of geographic location were limited to the information in user profiles, which may lack accuracy. Our analysis is also limited to the coders’ perceptions of user and audience race, gender identity, and sexual orientation. While we could have used a computer algorithm for coding, we chose to use traditional manual qualitative data analysis methods to allow for interpretation of nuances and the coding of multimedia sources. Lastly, the scope of our analysis was limited to digital information from the internet, and it lacks other information, such as information shared as part of community outreach and events. Nonetheless, we collected a large, comprehensive, international sample of tweets over many years.

### Conclusion

This study of tweets about HIV PrEP directed at women on Twitter identified a relatively limited and declining body of content. Many tweets used positive and appropriate imagery inclusive of people of color. These findings point to several areas for improvement for future Twitter campaigns directed at women about PrEP.

## Appendix

Multimedia Appendix 1 User codebook.

## Abbreviations

API: application programing interface
HBM: Health Belief Model
LGBTQ: lesbian, gay, bisexual, transgender, queer
MSM: men who have sex with men
PrEP: pre-exposure prophylaxis

## Data Availability

The data sets generated during and/or analyzed during the current study are available from the corresponding author upon request.

## References

[1] Basic Statistics | HIV Basics. Centers for Disease Control and Prevention.

[2] HIV and Women: HIV Diagnoses. Centers for Disease Control and Prevention.

[3] HIV and Women: PrEP Coverage. Centers for Disease Control and Prevention.

[4] Keddem S, Dichter ME, Hamilton AB, Chhatre S, Sonalkar S (2021). Awareness of HIV preexposure prophylaxis among people at risk for HIV: results from the 2017-2019 National Survey of Family Growth. Sex Transm Dis.

[5] Goparaju L, Experton LS, Praschan NC, Warren-Jeanpiere Lari, Young Mary A, Kassaye Seble (2015). Women want pre-exposure prophylaxis but are advised against it by their HIV-positive counterparts. J AIDS Clin Res.

[6] Sales JM, Sheth AN (2019). Associations among perceived HIV risk, behavioral risk and interest in PrEP among Black women in the Southern US. AIDS Behav.

[7] Hirschhorn Lisa R, Brown Rayna N, Friedman Eleanor E, Greene George J, Bender Alvie, Christeller Catherine, Bouris Alida, Johnson Amy K, Pickett Jim, Modali Laxmi, Ridgway Jessica P (2020). Black cisgender women's PrEP knowledge, attitudes, preferences, and experience in Chicago. J Acquir Immune Defic Syndr.

[8] Philbin MM, Parish C, Kinnard EN, Reed SE, Kerrigan D, Alcaide ML, Cohen MH, Sosanya O, Sheth AN, Adimora AA, Cocohoba J, Goparaju L, Golub ET, Fischl M, Metsch LR (2021). Interest in long-acting injectable pre-exposure prophylaxis (LAI PrEP) among women in the Women's Interagency HIV Study (WIHS): a qualitative study across six cities in the United States. AIDS Behav.

[9] Noar SM, Palmgreen P, Chabot M, Dobransky N, Zimmerman RS (2009). A 10-year systematic review of HIV/AIDS mass communication campaigns: Have we made progress?. J Health Commun.

[10] Phillips Gregory, Raman Anand B, Felt Dylan, McCuskey David J, Hayford Christina S, Pickett Jim, Lindeman Peter T, Mustanski Brian (2020). PrEP4Love: the role of messaging and prevention advocacy in PrEP attitudes, perceptions, and uptake among YMSM and transgender women. J Acquir Immune Defic Syndr.

[11] McLaughlin ML, Hou J, Meng J, Hu C, An Z, Park M, Nam Y (2016). Propagation of information about preexposure prophylaxis (PrEP) for HIV prevention through Twitter. Health Commun.

[12] Chen K, Duan Z, Yang S (2023). Twitter as research data: Tools, costs, skill sets, and lessons learned. Politics Life Sci.

[13] Kudrati SZ, Hayashi K, Taggart T (2021). AIDS Behav.

[14] Kakalou C, Lazarus JV, Koutkias V (2019). Mining social media for perceptions and trends on HIV pre-exposure prophylaxis. Stud Health Technol Inform.

[15] Schwartz J, Grimm J (2017). PrEP on Twitter: information, barriers, and stigma. Health Commun.

[16] Barrie C, Ho J (2021). academictwitteR: an R package to access the Twitter Academic Research Product Track v2 API endpoint. J Open Source Softw.

[17] Glanz K, Lewis FM, Rimer BK (1991). Health behavior and health education: Theory, research, and practice. Med Sci Sports Exerc.

[18] Jones CL, Jensen JD, Scherr CL, Brown NR, Christy K, Weaver J (2015). The Health Belief Model as an explanatory framework in communication research: exploring parallel, serial, and moderated mediation. Health Commun.

[19] Lowe A, Norris AC, Farris AJ, Babbage DR (2018). Quantifying thematic saturation in qualitative data analysis. Field Methods.

[20] Bos W, Tarnai C (1999). Content analysis in empirical social research. Int J Educ Res.

[21] Luca NR, Suggs LS (2013). Theory and model use in social marketing health interventions. J Health Commun.

[22] Willmott TJ, Rundle-Thiele S (2022). Improving theory use in social marketing: the TITE four-step theory application process. J Soc Mark.

[23] Hall KS (2012). The Health Belief Model can guide modern contraceptive behavior research and practice. J Midwifery Womens Health.

[24] Becker MH, Haefner DP, Kasl SV, Kirscht J P, Maiman LA, Rosenstock IM (1977). Selected psychosocial models and correlates of individual health-related behaviors. Med Care.

[25] Leonardo Z (2004). The color of supremacy: Beyond the discourse of ‘white privilege’. Educ Philos Theory.

[26] Rosenstock Gonzalez YR, Williams D, Herbenick D (2022). Skin color and skin tone diversity in human sexuality textbook anatomical diagrams. J Sex Marital Ther.

[27] Townes A, Guerra-Reyes L, Murray M, Rosenberg M, Wright B, Long L, Herbenick D (2020). 'Somebody that looks like me' matters: a qualitative study of black women's preferences for receiving sexual health services in the USA. Cult Health Sex.

[28] Harris JK, Choucair B, Maier RC, Jolani N, Bernhardt JM (2014). Are public health organizations tweeting to the choir? Understanding local health department Twitter followership. J Med Internet Res.

[29] Portman M (2017). Pre-exposure prophylaxis: making history. Sex Transm Infect.

[30] Alcantar Heredia JL, Goldklank S (2021). The relevance of pre-exposure prophylaxis in gay men's lives and their motivations to use it: a qualitative study. BMC Public Health.

[31] Mandavilli A (2023). H.I.V. Infections Remain Persistently High, U.N. Reports. New York Times.

[32] Mayer KH, Molina J, Thompson MA, Anderson PL, Mounzer KC, De Wet JJ, DeJesus E, Jessen H, Grant RM, Ruane PJ, Wong P, Ebrahimi R, Zhong L, Mathias A, Callebaut C, Collins SE, Das M, McCallister S, Brainard DM, Brinson C, Clarke A, Coll P, Post FA, Hare CB (2020). Emtricitabine and tenofovir alafenamide vs emtricitabine and tenofovir disoproxil fumarate for HIV pre-exposure prophylaxis (DISCOVER): primary results from a randomised, double-blind, multicentre, active-controlled, phase 3, non-inferiority trial. Lancet.

[33] Baldwin A, Light B, Allison WE (2021). Pre-exposure prophylaxis (PrEP) for HIV infection in cisgender and transgender women in the U.S.: A narrative review of the literature. Arch Sex Behav.

