# Supplementary material for: Characterizing Twitter Content About HIV Pre-exposure Prophylaxis (PrEP) for Women: Qualitative Content Analysis
Source: J Med Internet Res. 2023 May 11;25:e43596. doi: 10.2196/43596 (PMC10214116; doi:10.2196/43596)
Supplement: Multimedia Appendix 1 [file jmir_v25i1e43596_app1.docx]

**Multimedia Appendix 1. User Codebook**

| **Domain** | **Categories** | **Description/Examples** |
| --- | --- | --- |
| Coder Initials  *(Choose 1)* | - AA - AB - MK - SK - SM | - Identify coder |
| Twitter username  (*Choose 1*) | - Username/Hyperlink (Fill in) ______ - Deactivated user | - Fill in tweeter’s username. If account no longer available, select deactivated user |
| User type  *(Choose 1)* | - Individual – health professional - Individual – researcher/academic - Individual – activist/advocate - Individual – other (Fill in) ______ - Organization – healthcare - Organization – advocacy/NPO - Organization – general (Fill in) _____ - Undetermined | - Ex: HIV/STI doctor, nurse - Ex: Professor, epidemiologist - Ex: HIV activist, peer mentor - Ex: Author, artist, journalist - Ex: Healthcare services, HIV testing - Ex: AIDS institutes, local initiatives - Ex: Marketing, public relations - Name/description/profile unclear |
| User location  *(Choose 1)* | - US   - Select location - UK (Fill in) __________ - Brazil - Netherlands - Other (Fill in) ________ - None provided | If US selected, then choose one of below:  • General US - Ex: CDC, NIH  • Los Angeles – Ex: “Get0PrEPLA”  • DC – Ex: “PrEP4Her”  • New York  • Chicago  • Boston  • San Francisco  • Miami  • Other – (Fill in) ________ |
| Perceived gender identity/expression of individual user  *(Choose 1)* | - Female - Male - Other (Fill in) ________ - Not determined | - If other is selected, fill in the blank - Ex: they/them; non-binary, trans, etc. |
| Perceived race/ethnicity of individual user *(Choose 1)* | - POC - White - Black - Hispanic - Other (Fill in) _______ - Not determined | - Only select Black or Hispanic if explicitly stated by user, else use POC |
| Other individual user characteristics  *(Choose all that apply)* | - Identifies as LGBTQ+ - HIV/AIDS specific work - Personal account - Professional account - Other (Fill in) ______ | - Identify additional characteristics of the individual user |

**Tweet Codebook**

| **Domain** | **Categories** | **Description/Examples** |
| --- | --- | --- |
| Coder Initials  *(Choose 1)* | - AA - AB - MK - SK - SM | - Identify coder |
| Tweet ID *(Fill in)* | - Tweet ID/Hyperlink ______ | - Fill in the tweet’s hyperlink |
| Relevance to PrEP for women (includes cis + trans women)  *(Choose 1)* | - Yes - No, not about women - No, not about PrEP - Don’t know (Please explain) _____ | - Specifically mentions PrEP and features women. Includes transgender women. - Does not specifically mention and/or feature women - Not about PrEP - Ex: PEP, HIV treatment - Relevance of tweet cannot be determined. If selected, please explain   **** If No/Don’t know selected coding ends*** |
| Media type *(Choose 1)* | - Image + text - Image only - Tweet only (No image/video) - Video (Complete additional items) | - Text with visualization (bus ad, person) - Image with no text or slogans - Text only. No media included - If video is selected, choose one of below: - TED-style talk - Interview - formal - Marketing videos - Community interview/story - Animation - Other – (Fill in) _________ |
| Link contents  *(Choose all that apply)* | - Informational website (copy link below) ______________ - Tweet from another user WITH study hashtags - Tweet from another user WITHOUT study hashtags (copy link _______) - External video - News article - Document - Instagram/Facebook post - Research article - Other (Fill in) ________ - Broken Link - None | - If informational website is selected, copy the website link in the space provided - If tweet includes study hashtags, do not code the linked tweet - If selected, copy the tweet link in space provided and continue coding - YouTube, Facebook event video, film - Media related articles on PrEP - PDF or Word documents - Social media accounts - Manuscripts, peer reviewed publications - Specify the link’s purpose - Select if link does not work - No link is included in tweet |
| Imagery/visualization *(Choose all that apply)* | - None - PrEP pill/medication bottle - Symbolism / iconography - Logo/brand name - Marketing/promotional materials - Web Address URL (in image) - Contact information - Healthcare clinic setting - Locally relevant scenery - Campaign slogan (Fill in) ______ - Photograph - Other (Fill in) ________ - Person present | - No imagery in the tweet - Image of pill or medication present - Fist in air, medical symbol - Campaign logo, medication logo - Bus ads, pamphlets, T-shirt w/ slogans - Imagery includes web address - Imagery has phone #, address, email - Medical office, planned parenthood, etc. - Local skyline, barber shop, street view - Specify slogan. Excludes study hashtags - Pictures, Ex: Conference, photoshoots - Specify imagery - If selected complete **next 4 questions** |
| ***IF person present:***  Number of people present *(Choose 1)* | - 1 - 2-9 - 10+ | - Select number of people present in imagery |
| ***IF person present:***  Perceived gender identity/expression of individual user  *(Choose 1)* | - Female - Male - Other (Fill in) ________ - Not determined | - If other is selected, fill in the blank (Ex: they/them; non-binary, trans, etc.) |
| ***IF person present:***  Perceived race/ethnicity of individual user *(Choose 1)* | - POC - White - Black - Hispanic - Other (Fill in) _______ - Not determined | - Only select Black or Hispanic if explicitly stated by user, else use POC |
| ***IF person present:***  Other individual user characteristics  *(Choose all that apply)* | - Cartoon character/mascot - Healthcare worker - Journalist - LGBTQ+ - Other (Fill in) ______ | - Identify additional characteristics of the person(s) present in the imagery |
| Health beliefs  (*Choose all that apply)* | - Risk of HIV/AIDS - Benefits of PrEP - Self-efficacy/empowerment - Cues to action - Barriers to PrEP - None | - HIV/AIDS severity/susceptibility - Ex: 1 in 5 new HIV diagnoses in US are women. - Ex: PrEP is a daily medication taken orally to reduce your risk of HIV. - Ex: "PrEP puts me in charge - I'm making sure I'm in control" - Concrete actionable items – Ex: “Go see Dr. NOT: “Go to event” - Ex: You don’t have to go to the clinic to receive PrEP at home! - Select if no health belief constructs present |
| Purpose  (*Choose all that apply)* | - Raising awareness of PrEP - Promoting PrEP use and access - Sharing information/resources - Providing event details - Personal narrative/experience - Soliciting audience engagement - Fundraising - Merchandise - Other (Fill in) ___________ - None | - Must mention term “awareness” of PrEP - Ex: #DYK that #PrEP is for women too? - Ex: Now offering PrEP at X location - Info on upcoming events, health fairs, etc - Individuals sharing PrEP use stories - Ex: use locator to find PrEP resources - Ex: PrEP charity run - PrEP T-shirts, tank tops/vests w/ logos - Specify other purpose - Select if no clear purpose determinable from tweet. |
| Target audience  (*Choose all that apply*) | - Women - People of color - Black people - White people - Health professionals - Spanish speaking - Parents/mothers - Gay/lesbian/bisexual/queer - Transgender women - Sero-discordant couples - Heterosexual couples - Other (Fill in) ________ - None/undetermined | - Select specific target audiences mentioned in tweet. - Specify target audience if not in list - Select if no clear target audience |
| Additional notes  *(If applicable, fill in)* | - ______________________ | - Use this space to add any thoughts, ideas, or questions for discussion |
| Good tweet?  *(Choose one)* | - Yes (Explain why) ___________ - No | - If yes is selected, explain why tweet is a good example for use |

**Website Codebook**

| **Domain** | **Categories** | **Description/Examples** |
| --- | --- | --- |
| Coder Initials  *(Choose 1)* | - AA - AB - MK - SK - SM | - Identify coder |
| Website URL *(Fill in)* | - Informational website URL ______ | - Fill in the website hyperlink |
| Relevant to women and PrEP (includes cis + trans women)  *(Choose 1)* | - Yes - No, not about women - No, not about PrEP - Don’t know (Please explain) _____ | - Specifically mentions PrEP and features women. Includes transgender women - Does not specifically mention and/or feature women - Not about PrEP (Ex: PEP, HIV treatment) - Relevance of website cannot be determined. If selected, please explain   **** If No/Don’t know selected coding ends*** |
| Media type  *(Choose 1)* | - Image + text - Text only (No image/video) - Contains video (Complete additional items) | - Text with visualizations (Ex. PrEP users) - Text only. No media included - If video is selected, choose one of below: - TED-style talk - Interview - formal - Marketing videos - Community interview/story - Animation - Other – (Fill in) _________ |
| Imagery/visualization *(Choose all that apply)* | - None - PrEP pill/medication bottle - Symbolism / iconography - Logo/brand name - Marketing/promotional materials - Web Address URL (in image) - Contact information - Healthcare clinic setting - Locally relevant scenery - Campaign slogan (Fill in) ______ - Photograph - Conference presentations/materials - Other (Fill in) ________ - Person present | - No imagery in website - Image of pill or medication present - Fist in air, medical symbol - Campaign logo, medication logo - Pamphlets, T-shirt w/ slogans - Site includes additional web addresses - Site has phone #, address, email - Medical office, planned parenthood, etc. - Local skyline, barber shop, street view - Specify slogan. Excludes study hashtags - Pictures. Ex: Conference, photoshoots - Slides, handouts - Specify imagery - If selected complete **next 4 questions** |
| ***IF person present:***  Number of people present *(Choose 1)* | - 1 - 2-9 - 10+ | - Select number of people present on the site’s home page |
| ***IF person present:***  Perceived gender identity/expression of individual user  *(Choose 1)* | - Female - Male - Other (Fill in) ________ - Not determined | - If other is selected, fill in the blank (Ex: non-binary, third gender, etc.) |
| ***IF person present:***  Perceived race/ethnicity of individual user *(Choose 1)* | - POC - White - Black - Hispanic/Latinx - Not determined | - Only select Black or Hispanic if explicitly stated on site, else use POC |
| ***IF person present:***  Other individual user characteristics  *(Choose all that apply*) | - Cartoon character/mascot - Healthcare worker - Journalist - LGBTQ+ - Other (Fill in) ______ | - Identify additional characteristics of the person(s) present on the site |
| Health beliefs  *(Choose all that apply)* | - Risk of HIV/AIDS - Benefits of PrEP - Self-efficacy/empowerment - Cues to action - Barriers to PrEP - None | - HIV/AIDS severity/susceptibility - Ex: 1 in 5 new HIV diagnoses in US are women. - Ex: PrEP is a daily medication taken orally to reduce your risk of HIV. - Ex: "PrEP puts me in charge - I'm making sure I'm in control" - Concrete actionable items, Ex: “Go see Dr. NOT: “Go to event” - Ex: You don’t have to go to the clinic to receive PrEP at home! - Select if no health belief constructs present on the site |
| Purpose  (*Choose all that apply)* | - Raising awareness of PrEP - Promoting PrEP use and access - Sharing information/resources - Providing event details - Personal narrative/experience - Soliciting audience engagement - Fundraising - Merchandise - Other (Fill in) ___________ - None | - Must mention term “awareness” of PrEP - Ex: #DYK that #PrEP is for women too? - Ex: Now offering PrEP at X location - Info on upcoming events, health fairs, etc - Individuals sharing PrEP use stories - Use this locator to find PrEP resources - Ex: PrEP charity run - PrEP T-shirts, tank tops/vests w/ logos - Specify other purpose of website - Select if no clear purpose determinable from website |
| Target audience  (*Choose all that apply*) | - Women - People of color - Black people - White people - Health professionals - Spanish speakers - Parents/mothers - Gay/lesbian/bisexual/queer - Transgender women - Sero-discordant couples - Heterosexual couples - Pregnant wome - Intravenous drug users - Other (Fill in) ________ - None/undetermined | - Select specific target audiences mentioned on the website - Specify target audience if not in list - Select if no clear target audience |
| Additional notes  *(If applicable, fill in)* | - ______________________ | - Use this space to add any thoughts, ideas, or questions for discussion |
| Good website?  *(Choose 1)* | - Yes (Explain why) ___________ - No | - If yes is selected, explain why website is a good example for use |
| Link Type  *(Choose all that apply)* | - Informational website - Twitter URL - YouTube video - News article - PDF - Facebook page - Research article - Broken Link | - Web link is to an informational website - Web link is to another twitter URL - Web link is to a YouTube video - Web link is to articles on PrEP - Web link is a PDF - Web link is to FB social media accounts - Links to manuscripts, journals - Link does not work |
| Link outs  *(Fill in)* | - Link out descriptions ____________ | - IF home page has additional links, describe their purpose. Ex: Links to CDC, PrEP/HIV resources, slides, articles, vids |
| News article title  *(Fill in)* | - News title ___________ | - For links to news articles, copy the title of the article in the space provided |
